
JOURNAL INFORMATION
==============================
NLM Title Abbreviation: J Vet Intern Med
Journal ID: J Vet Intern Med
NLM Journal ID: 8708660
Journal ID: JVIM

Journal of Veterinary Internal Medicine

ISSN: 0891-6640
EISSN: 1939-1676
Publisher: Oxford University Press

ARTICLE INFORMATION
==============================
PMCID: PMC11474585
DOI: 10.1111/jvim.16460
Article ID: JVIM16460
Article version: 1
Subjects: Issue Information, Issue Information

Issue Information

Electronic publication date: 2023 Jul 24
Publication date: 2023 Jul-Aug
Volume: 37
Issue: 4
First page: 1289
Last page: 1295
Copyright: © 2023 The Authors. Journal of Veterinary Internal Medicine published by Wiley Periodicals LLC on behalf of American College of Veterinary Internal Medicine.
License: This is an open access article under the terms of the http://creativecommons.org/licenses/by/4.0/ License, which permits use, distribution and reproduction in any medium, provided the original work is properly cited.
License URL: https://creativecommons.org/licenses/by/4.0/

Figure count: 0
Table count: 0
Page count: 1

source-schema-version-number: 2.0
cover-date: July/August 2023
details-of-publishers-convertor: Converter:WILEY_ML3GV2_TO_JATSPMC version:6.4.9 mode:remove_FC converted:15.10.2024

==============================

Volume 37, Number 4 July/August 2023

### JOURNAL OF VETERINARY INTERNAL MEDICINE

Open Access

ACVIM
American College of Veterinary Internal Medicine

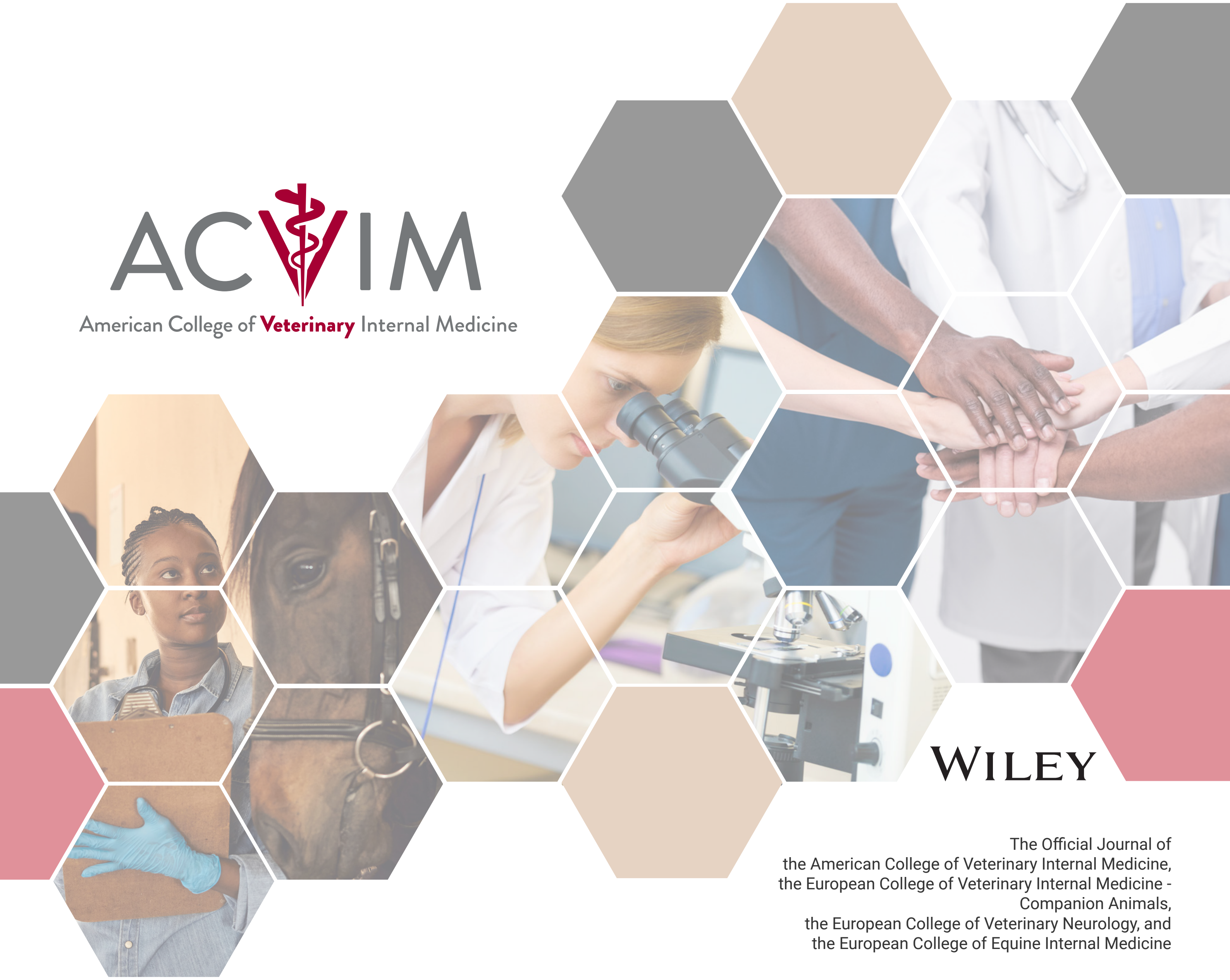

WILEY

The Official Journal of
the American College of Veterinary Internal Medicine,
the European College of Veterinary Internal Medicine - Companion Animals,
the European College of Veterinary Neurology, and
the European College of Equine Internal Medicine

### Journal of Veterinary Internal Medicine

Open Access

ACVIM American College of Veterinary Internal Medicine

The Official Journal of the American College of Veterinary Internal Medicine,
the European College of Veterinary Internal Medicine - Companion Animals, the European College of Veterinary Neurology, and the European College of Equine Internal Medicine

**MISSION STATEMENT**

The mission of the *Journal of Veterinary Internal Medicine* is to advance veterinary medical knowledge and improve the lives of animals by publication of authoritative scientific articles of animal diseases.

**Volume 37, Number 4** **July/August 2023**

LETTERS TO THE EDITOR

1296 **Letter regarding "Validation of a cage-side agglutination card for *Dal* blood typing in dogs" – Validation of agglutination card kit or simple comparison to gel column method for canine *Dal* blood typing?**
*Urs Giger and Kathleen P. Freeman*

1298 **Response to letter regarding "Validation of a cage-side agglutination card for *Dal* blood typing in dogs"**
*Marie-Claude Blais and Emilie Véran*

SMALL ANIMAL

Review Articles

*Gastroenterology*

1299 **A 20-year scoping review of the veterinary interventional radiology and interventional endoscopy literature (2000-2019)**
*Nina Samuel, Michelle A. Giuffrida, William T. N. Culp, and Carrie A. Palm*

*Neurology*

1306 **Phenotypic and genetic aspects of hereditary ataxia in dogs**
*Kimberley Stee, Mario Van Poucke, Mark Lowrie, Luc Van Ham, Luc Peelman, Natasha Olby, and Sofie F.M. Bhatti*

Standard Articles

*Cardiology*

1323 **Effect of a specially formulated diet on progression of heart enlargement in dogs with subclinical degenerative mitral valve disease**
*Mark A. Oyama, Brian A. Scansen, Adrian Boswood, Guilherme Goldfeder, Steven Rosenthal, Richard Cober, Kendra LaFauci, Ryan C. Friese, Márcia Gomes, Yu-Mei Ruby Chang, and Qinghong Li*

*Endocrinology*

1331 **Effect of pituitary-dependent hypercortisolism on the survival of dogs treated with radiotherapy for pituitary macroadenomas**
*Sofia Rapastella, Simona Morabito, Mellora Sharman, Jérôme Benoit, Luca Schiavo, Joanna Morris, Jane Margaret Dobson, and Christopher John Scudder*

1341 **Evaluation of oral telmisartan administration as a suppression test for diagnosis of primary hyperaldosteronism in cats**
*Virginie Fabrès, Renaud Dumont, Mélanie Garcia, Dan Rosenberg, Benoit Rannou, Maxine Kurtz, and Ghita Benchekroun*

1348 **Prospective evaluation of a telmisartan suppression test as a diagnostic tool for primary hyperaldosteronism in cats**
*Maxime Kurtz, Virginie Fabrès, Renaud Dumont, Valérie Chetboul, Sabine Chahory, Vittorio Saponaro, Emilie Trehiou, Camille Poissonnier, Peggy Passavin, Coline Jondeau, Matthieu Bott, Thierry Buronfosse, and Ghita Benchekroun*

*Gastroenterology*

1358 **Serum proteome profiles in cats with chronic enteropathies**
*Jane Yu, Lara Boland, Melissa Catt, Leah Puk, Nadia Wong, Mark Krockenberger, Peter Bennett, Craig Ruaux, and Valerie C. Wasinger*

1368 **Serum cobalamin and methylmalonic acid concentrations in juvenile dogs with parvoviral enteritis or other acute enteropathies**
*Michael Hung, Justin Heinz, Jörg M. Steiner, Jan Suchodolski, and Jonathan Lidbury*

*Hematology*

1376 **Cephalic and saphenous venous blood collected by continuous heating of the paws compared with arterial blood for measurement of blood gas values in well-perfused dogs**
*Yukihito Shiroshita, Ryou Tanaka, Miki Shimizu, and Yoshihisa Yamane*

1390 **Synergistic inhibitory effects of clopidogrel and rivaroxaban on platelet function and platelet-dependent thrombin generation in cats**
*Sara T. Lo, Ronald H. L. Li, Catherine J. Georges, Nghi Nguyen, Cheyenne K. Chen, Claire Stuhlmann, Maureen Sigmund Oldach, Victor Noel Rivas, Samantha Fousse, Samantha P. Harris, and Joshua A. Stern*

Nephrology/Urology

1401 **Comparison of cystocentesis versus home sampling to determine urinary protein: Creatinine ratio and urine specific gravity in cats**
*Femke Mortier, Sylvie Daminet, Luc Duchateau, Sofie J. J. Marynissen, and Dominique Paepe*

*Neurology*

1409 **Investigation of the presence of specific neural antibodies in dogs with epilepsy or dyskinesia using murine and human assays**
*Lea Hemmeter, Christian G. Bien, Corinna I. Bien, Andrea Tipold, Jasmin Neßler, Andrea Bathen-Nöthen, Kaspar Matiasek, Maik Dahlhoff, Clare Rusbridge, Carina Rotter Black, Kai Rentmeister, Holger A. Volk, and Andrea Fischer*

1418 **Magnetic resonance imaging and neurologic characterization of combined osseous- and disc-associated cervical spondylomyelopathy in dogs**
*Marília de Albuquerque Bonelli and Ronaldo Casimiro da Costa*

1428 **Clinical presentation, diagnosis, treatment, and outcome in 8 dogs and 2 cats with global hypoxic-ischemic brain injury (2010-2022)**
*Abbe Harper Crawford, Elsa Beltran, Cecilia-Gabriella Danciu, and Dylan Yaffy*

1438 **Clinical features, comparative imaging findings, treatment, and outcome in dogs with discospondylitis: A multi-institutional retrospective study**
*Cassie Van Hoof, Nicole A. Davis, Sheila Carrera-Justiz, Alisha D. Kahn, Steven De Decker, Nicholas J. Grapes, Michaela Beasley, John Du, Theresa E. Pancotto, Anna Suñol, Richard Shinn, Barry DeCicco, Erica Burkland, and Harry Cridge*

1447 **The T2-FLAIR mismatch sign as an imaging biomarker for oligodendrogliomas in dogs**
*Josefa Garcia-Mora, Rell L. Parker, Thomas Cecere, John L. Robertson, and John H. Rossmeisl*

*Oncology*

1455 **Therapeutic response and prognostic factors of 14 dogs undergoing transcatheter arterial embolization for hepatocellular masses: A retrospective study**
*Yuta Kawamura, Hiroki Itou, Akitomo Kida, Hiroki Sunakawa, Moe Suzuki, and Kenji Kawamura*

1466 **Prognosis of primary pulmonary adenocarcinoma after surgical resection in small-breed dogs: 52 cases (2005-2021)**
*Masanao Ichimata, Yumiko Kagawa, Keita Namiki, Atsushi Toshima, Yuko Nakano, Fukiko Matsuyama, Eri Fukazawa, Kei Harada, Ryuzo Katayama, and Tetsuya Kobayashi*

*Respiratory*

1475 **Description of a novel method for detection of sleep-disordered breathing in brachycephalic dogs**
*Iida Niinikoski, Sari-Leena Himanen, Mirja Tenhunen, Liisa Lilja-Maula, and Minna M. Rajamäki*

Case Reports

*Cardiology*

1482 **Right atrial thrombus formation in a dog after successful electrical cardioversion for atrial fibrillation**
*Marta Karn, Brianna M. Potter, Brian A. Scansen, June A. Boon, and Viktor Szatmári*

*Nephrology/Urology*

1488 **Urinary bladder hemangiosarcoma in a cat treated with partial cystectomy and adjuvant metronomic cyclophosphamide and thalidomide**
*Abigail McNally, Matteo Rossanese, Alejandro Suárez-Bonnet, Alexandros Hardas, and Andrew D. Yale*

*Neurology*

1493 **Hypertensive nonobstructive hydrocephalus as main magnetic resonance imaging feature in a dog with disseminated choroid plexus carcinomatosis**
*Lea Carisch, Lorenzo Golini, Lea Schurna, Chiara Bergamino, Katrin M. Beckmann, Monika Hilbe, Thanaporn Asawapattanakul, Wolfgang Baumgärtner, Christina Puff, and Adriano Wang-Leandro*

1501 **A novel meningioma with tyrosine-rich crystals in a 6-year-old Great Dane**
*Kara Majors, Savannah M. Rocha, Rebecca Windsor, Ronald B. Tjalkens, Julia Engelien, and Tawfik Aboellail*

*Nutrition*

1507 **Mutations in the *CYP27B1* gene cause vitamin D dependent rickets in pugs**
*Cecilia Rohdin, Chao Wang, Gustaf Brander, Veronica Rondahl, Åsa Karlsson, Lisa Friling, Anthony Fischetti, Jennifer Meadows, Jens Häggström, Karin Hultin Jäderlund, Ingrid Ljungvall, and Kerstin Lindblad-Toh*

**EQUINE**

Standard Articles

*Exercise Physiology*

1514 **Medical causes of poor performance and their associations with fitness in Standardbred racehorses**
*Chiara M. Lo Feudo, Luca Stucchi, Bianca Conturba, Giovanni Stancari, Enrica Zucca, and Francesco Ferrucci*

*Gastroenterology*

1528 **Effect of diet composition on glandular gastric disease in horses**
*Samy Julliand, Marjorie Buttet, Tanguy Hermange, Patrick Hillon, and Véronique Julliand*

1537 **Evaluation of the effects of medium-term (57-day) omeprazole administration and of omeprazole discontinuation on serum gastrin and serum chromogranin A concentrations in the horse**
*Bethanie Clark, Catherine Steel, Jessica Vokes, Jack R. Shan, Kristene Gedye, Amy Lovett, and B. W. Sykes*

1544 **Clinical presentation and outcome of gastric impactions with or without concurrent intestinal lesions in horses**
*Sophie E. Talbot, Rose Tallon, and Bettina Dunkel*

*Hematology*

1552 **Red blood cell distribution width to platelet ratio in neonatal foals with sepsis**
*Rebeca Scalco, Gabriela Novo de Oliveira, Bruna da Rosa Curcio, McCaide Wooten, K. Gary Magdesian, Stephanie Takako Hidai, Pranav Pandit, and Monica Aleman*

*Infectious Disease*

1561 **Efficacy of high-level disinfection of endoscopes contaminated with *Streptococcus equi* subspecies *equi* with 2 different disinfectants**
*Veridiana Nadruz, Laurie A. Beard, Katherine M. Delph-Miller, Robert L. Larson, Jianfa Bai, and Muckatira M. Chengappa*

*Neurology*
1568 **Cerebellar axonopathy in Shivers horses identified by spatial transcriptomic and proteomic analyses**
*Stephanie J. Valberg, Zoë J. Williams, Marisa L. Henry, and Carrie J. Finno*

**FOOD ANIMAL**

Standard Articles

*Gastroenterology*
1580 **Continuous fluid infusion per rectum compared with intravenous fluid infusion in pigs**
*Munashe Chigerwe, Sarah J. Blasczynski, Bailey A. Abi-Nader, Paige M. Condy, Cileah M. Kretsch, and Sarah M. Depenbrock*

1588 **Effect of time of sample collection after onset of diarrhea on fecal microbiota composition of calves**
*Lynna Li, David L. Renaud, Hanne M. Goetz, Emma Jessop, Marcio C. Costa, Lisa Gamsjäger, and Diego E. Gomez*

*Hematology*
1594 **Agglutination and hemolytic crossmatching to determine transfusion reaction differences between large and small breed goats**
*Cileah M. Kretsch, Flavio H. Alonso, Maggie Buktenica, and Meera C. Heller*

*Respiratory*
1603 **Bayesian evaluation of the accuracy of a thoracic auscultation scoring system in dairy calves with bronchopneumonia using a standard lung sound nomenclature**
*Antonio Boccardo, Salvatore Ferraro, Giulia Sala, Vincenzo Ferrulli, Davide Pravettoni, and Sébastien Buczinski*

Case Report

*Gastroenterology*
1614 **Acute cysticercosis caused by *Cysticercus tenuicollis* in lambs**
*Mostafa Abdollahi, Samad Lotfollahzadeh, Sara Shokrpoor, and Iradj Ashrafi Tamai*

### Journal of Veterinary Internal Medicine

Open Access

ACVIM American College of Veterinary Internal Medicine

The Official Journal of the American College of Veterinary Internal Medicine,
the European College of Veterinary Internal Medicine - Companion Animals, the European College of Veterinary Neurology, and the European College of Equine Internal Medicine

### EDITORIAL BOARD

Kenneth W. Hinchcliff, BVSc, PhD, DACVIM
Editor-in-Chief
The University of Melbourne
Stephen P. DiBartola, DVM, DACVIM
Editor-in-Chief
The Ohio State University
Joshua Stern, DVM, PhD, DACVIM
Associate Editor - Cardiology
University of California, Davis
Katharyn Mitchell, BVSc, DipVetClinStud, DVM, PhD, DACVIM
Associate Editor - Large Animal Internal Medicine (Equine, Cardiology)
Cornell University
Sébastien M. C. Buczinski, DMV, MSc, DACVIM
Consulting Editor for Experimental Design & Statistics
University of Montreal
George Moore, DVM, MS, PhD, DACVIM
Consulting Editor for Experimental Design & Statistics
Purdue University
Kristopher J. Hughes, BVSc (Hons 1), FANZCVS, DECEIM, Associate ACVIM
ECEIM Associate Editor - Large Animal Internal Medicine (Equine)
Charles Sturt University
Linda Kidd, DVM, PhD, DACVIM
Associate Editor - Small Animal Internal Medicine (Infectious Disease)
Linda Kidd Veterinary Internal Medicine Consulting
M. Julia B. Felippe, MV, MSc, PhD, DACVIM
Associate Editor - Large Animal Internal Medicine (Equine)
Cornell University
Shelly L. Vaden, DVM, PhD, DACVIM
Associate Editor - Small Animal Internal Medicine (Nephrology/Urology)
North Carolina State University
Michael Willard, DVM, DACVIM
Associate Editor - Small Animal Internal Medicine
Texas A&M University
Andrea Tipold, DVM, DECVN
ECVN Associate Editor - Neurology
School of Veterinary Medicine Hannover
Robert Rebhun, DVM, PhD, DACVIM
Associate Editor - Hematology/Oncology
University of California, Davis
Eric Zini, PD, PhD, DECVIM-CA
ECVIM-CA Associate Editor - Small Animal Internal Medicine (Endocrinology)
University of Zurich & Istituto Veterinario di Novara & University of Padua
Munashe Chigerwe, BVSc, MPH, PhD, DACVIM
Associate Editor - Large Animal Internal Medicine (Food & Fiber)
University of California, Davis
Lauren Trepanier, DVM, PhD, DACVCP, DACVIM
Associate Editor – Small Animal Internal Medicine (Pharmacology, Pharmacokinetics, Toxicology, and Therapeutic Trials)
University of Wisconsin

### ACVIM Board of Regents Officers

Jane Sykes, BVSc (Hons), PhD, MBA, DACVIM
Board Chairperson
Joerg Steiner, DMV, PhD, DACVIM, DECVIM-CA
President
Bill Tyrrell, DVM, DACVIM
Treasurer

### Ex-Officio Member of the ACVIM Board of Regents

Harold McKenzie, DVM, MS, DACVIM
Immediate Past Board Chair

### The Editorial and ACVIM Offices are located at:

5500 Greenwood Plaza Boulevard, Suite 130, Greenwood Village, CO 80111

**Telephone:** 303-231-9933 or 800-245-9081

**Editorial Email:** JVIM@ACVIM.org.
**ACVIM Email:** ACVIM@ACVIM.org.

The Journal is available online at www.wileyonlinelibrary.com/journal/jvim.

### ACVIM Board of Regents Members

Sandra D. Taylor, DVM, PhD, DACVIM
Large Animal Internal Medicine At Large
Karyn Harrell, DVM, DACVIM
Small Animal Internal Medicine At Large
Phoebe A. Smith, DVM, DACVIM
Private Practice At Large
Roger A. Hostutler, DVM, MS, DACVIM
President, Small Animal Internal Medicine
Chris Sanchez, DVM, PhD, DACVIM
President, Large Animal Internal Medicine
Steven L. Rosenthal, DVM, DACVIM
President, Cardiology
Joan R. Coates, DVM, MS, DACVIM
President, Neurology
Chad Johannes, DVM, DACVIM
President, Oncology
Shannon Carter, EdD, CAE
Chief Executive Officer

### Journal of Veterinary Internal Medicine

Open Access

ACVIM American College of Veterinary Internal Medicine

The Official Journal of the American College of Veterinary Internal Medicine, the European College of Veterinary Internal Medicine - Companion Animals, the European College of Veterinary Neurology, and the European College of Equine Internal Medicine

**Disclaimer**
The Publisher, the American College of Veterinary Internal Medicine and Editors cannot be held responsible for errors or any consequences arising from the use of information contained in this journal; the views and opinions expressed do not necessarily reflect those of the Publisher, the American College of Veterinary Internal Medicine or Editors, neither does the publication of advertisements constitute any endorsement by the Publisher, the American College of Veterinary Internal Medicine or Editors of the products advertised.

**Open Access and Copyright**
Articles published by the *Journal of Veterinary Internal Medicine* are fully open access: immediately freely available to read, download and share. *Journal of Veterinary Internal Medicine* articles are published under the terms of the Creative Commons Attribution Non-Commercial License (CC BY NC), which permits use, distribution and reproduction in any medium, provided the original work is properly cited and is not used for commercial purposes.
Copyright on any research article published in the *Journal of Veterinary Internal Medicine* is retained by the author(s). Authors grant Wiley a license to publish the article and identify itself as the original publisher. Authors also grant any third party the right to use the article freely as long as its integrity is maintained and its original authors, citation details and publisher are identified. The Wiley Open Access Agreement formalizes these and other terms and conditions of publishing research articles.

**Use by commercial “for-profit” organizations**
Use of Wiley Open Access articles for commercial, promotional, or marketing purposes requires further explicit permission from Wiley and will be subject to a fee. All requests to republish the article (in whole or in part) in another work such as a book or journal article (other than normal quotations with an appropriate citation) can be cleared directly from the point of content using the automated RightsLink service. Simply click on the ‘Request Permission’ link to complete your order.
For other commercial, promotional, or marketing purposes please contact corporatesales@wiley.com. Commercial purposes include: Copying or downloading of articles, or linking to such articles for further redistribution, sale or licensing; Copying, downloading or posting by a site or service that incorporates advertising with such content; The inclusion or incorporation of article content in other works or services (for example, a compilation educational purposes; Use for the purposes of monetary reward by means of sale, resale, licence, loan, transfer or other form of commercial exploitation such as marketing products; Print reprints/eprints of Wiley Open Access articles.

*JOURNAL OF VETERINARY INTERNAL MEDICINE* (ISSN 1939-1676 [online]) is published bimonthly on behalf of the American College of Veterinary Internal Medicine by Wiley Periodicals LLC, 111 River St., Hoboken, NJ 07030-5774.

**Publisher**
*Journal of Veterinary Internal Medicine* is published by Wiley Periodicals LLC, 350 Main St., Malden, MA 02148-5020

**Production Editor:** Deepika Prakash (email: JVIM@wiley.com).

**Commercial Reprints:** Helane Daniels (email: hdaniels@wiley.com)

**Abstracting and Indexing Services**
The Journal is indexed by Science Citation Index, and MEDLINE. For a complete list of A&I Services visit: www.wilevonlinelibrarv.com/iournal/jvim

ISSN 1939-1676 (Online)

For submission instructions, and other information visit: www.wileyonlinelibrary.com/journal/jvim

**Manuscript Review**
All manuscripts are reviewed by experts in the field who advise the editors of the manuscript's scientific quality. Decisions regarding publication are made by the Co-Editors-in-Chief acting on the advice of reviewers and Associate Editors.

Wiley's Corporate Citizenship initiative seeks to address the environmental, social, economic, and ethical challenges faced in our business and which are important to our diverse stakeholder groups. Since launching the initiative, we have focused on sharing our content with those in need, enhancing community philanthropy, reducing our carbon impact, creating global guidelines and best practices for paper use, establishing a vendor code of ethics, and engaging our colleagues and other stakeholders in our efforts.

Follow our progress at www.wiley.com/go/citizenship
